# High urban tuberculosis case notification rates can be misleading: evidence from an urban setting in Ethiopia

**DOI:** 10.1186/s12889-020-8290-z

**Published:** 2020-03-06

**Authors:** Daniel Datiko, Ameha Hadgu, Degu Jerene, Pedro G. Suarez

**Affiliations:** 1 Challenge TB, Management Sciences for Health, Addis Ababa, Ethiopia; 2 Management Sciences for Health, Arlington, USA

**Keywords:** Urban, Cities, Case notification, Ethiopia

## Abstract

**Background:**

Tuberculosis (TB) is a major public health problem. Its magnitude the required interventions are affected by changes in socioeconomic condition and urbanization. Ethiopia is among the thirty high burden countries with increasing effort to end TB. We aimed to describe the case notification rate (CNR) for urban tuberculosis (TB) and estimate the percentage of TB patients who are not from the catchment population.

**Methods:**

This cross-sectional study used data from TB registers from 2014/15 to 2017/18. We calculated the CNR and treatment success rate for the study area.

**Results:**

Of 2892 TB cases registered, 2432 (84%) were from Adama City, while 460 (16%) were from other sites. The total TB CNR (including TB cases from Adama and other sites) was between 153 and 218 per 100,000 population. However, the adjusted TB CNR (excluding cases outside Adama City) was lower, between 135 and 179 per 100,000. Of 1737 TB cases registered, 1652 (95%) were successfully treated. About 16% of TB cases notified contributing to CNR of 32 per 100,000 population is contributed by TB cases coming from outside of Adama city.

The CNR of 32 per 100,000 population (ranging from 18 to 46 per 100,000) for Adama City was from the patients that came from the surrounding rural areas who sought care in the town.

**Conclusion:**

Although the TB CNR in Adama City was higher than the national CNR, about one-fifth of TB cases came from other sites-which led to overestimating the urban CNR and underestimating the CNR of neighboring areas. TB programs should disaggregate urban TB case notification data by place of residence to accurately identify the proportion of missed cases.

## Background

Tuberculosis (TB) is a disease deeply rooted in social fabrics that requires both a biomedical and social response. It has been long recognized as a disease whose transmission is favored by the density of urban populations [1–4]. TB is a disease of the poor, and its burden often serves as a marker of social inequality [5]. Although the effect of urbanization on TB burden is mixed [6], compared to rural areas, TB case notification rates (CNRs) in urban and peri-urban areas reflect high TB burden in urban areas [7]. This is due to underlying social and economic determinants of health that include not only poverty but also overcrowding, urban in-migration, deficient social protection, stigma and discrimination, HIV infection, and limited access to health services [8–10].

Given these socioeconomic problems, it is not a surprise that urban areas carry the highest burden of TB and notify more cases. Furthermore, the size of urban populations and socioeconomic problems are rapidly increasing in developing and high-TB-burden countries [3], which will increase the number of people exposed to high-risk environments for communicable diseases, including TB. Consequently, higher CNRs from urban areas are often taken as markers of higher disease burden, which has resulted in a tendency and commitment to direct more resources and target efforts to urban areas through ambitious major global Zero TB initiatives [6, 11]. However, other factors that contribute to higher TB case notification in cities or draw cases to cities are rarely considered. The “pull” factors that lead to seeking care in urban areas might influence the CNR. These factors might include the need for better health care, inadequacies in rural TB services, inaccessibility of services, patient preference, stigma and discrimination, limited case-finding interventions, shortage of diagnostic facilities, or inadequate technical capacity of health care workers. Despite these factors, in rural areas there has been limited effort to disaggregate TB patients to their place of residence and calculate the actual CNR by the source community for the notified TB cases. TB patients in peri-urban areas travel to seek care in urban facilities which compromises patient follow up. Outcomes for patients are, therefore, undermined; these include increased loss to follow-up, poor treatment adherence (due to inadequacies in referral linkage to treatment sites and the need for frequent travel to distant sites by patients), and unaffordable cost of seeking care. Because these patients come from other catchment areas, they are subject to more out-of-pocket expenditures and opportunity costs, which extend from the patient to their households or relatives [12, 13]. These factors contribute to making TB services prohibitively expensive and demand attention from National TB Programs (NTPs) [14–17].

On one hand, urban areas consistently have higher CNRs, more than the estimated TB burden in some cities [18]. The higher CNRs in urban areas, on the other hand, may lead to an inaccurate impression of higher success in terms of achieving targets for case finding and lead to reluctance, and reduction in efforts, to intensify case finding within the community. The NTP assumes equal incidence for urban and rural TB and reports cases by reporting site, not the address where patients live. To our knowledge, there has been no national review to see if urban areas have actually achieved targets or missed cases within their populations.

### Objective

The aim of this study was to describe urban CNR and estimate the percentage of TB patients who came from other catchment areas in the study area.

## Methods

### Study area and design

This was a cross-sectional study involving the analysis of secondary data from the unit TB registers in urban areas.

Ethiopia is among the 30 high-TB-burden countries [7]. TB diagnostic and treatment services are provided in health centers and hospitals. The End TB strategy has been adopted and the country is committed to reaching missed TB cases in the vulnerable population.

Adama is one of the urban areas in Oromia region, located about 100 km away from Addis Ababa, the capital of Ethiopia. It has a population of about 386,237 people living in the areas. The health service coverage has reached to 95% of the population. Adama city is surrounded by rural district and rural communities who dwell on farming. A total of 26 health facilities, one public hospital, two non-governmental organization (NGO) clinics, 8 public health centers, and 15 private facilities provide health services to the population in the area. All public and private health facilities provide TB screening services for their clients. Ninety-two percent of the health facilities provide diagnostic and treatment services while 77% provide diagnostic services. TB case finding and treatment outcome data were obtained from the TB registers of facilities in Adama City from 2014/15 to 2017/18. All TB patients that were registered and received treatment in the selected health facilities were included in the study.

### Data management

#### Data sources and variables

The variables used for the study include age, sex, place of residence, age, TB classification, TB category, and the treatment outcome. The data were obtained from unit TB registers in the health facilities. The outcome variables are case notification rate and treatment success rates.

#### Data collection and quality assurance

Health facilities providing TB diagnosis and treatment services record and report their quarterly case notification data and treatment outcomes from their unit TB registers. The data for this study were extracted from those registers. A senior data expert worked with the Oromia Zonal Cluster Coordinator and Adama City TB Program Coordinator, and TB focal persons collected the data. In discussion with the TB focal person in the health facilities, we have identified all TB registers that were used and currently in use for patient registration and follow up for the study period. All the data from the registers were entered into using trained data clerk loosely working with our senior data analyst. We used an electronic data capturing method. All TB unit registers containing lists of TB patients’ records for the study period were scanned. The data were transcribed by a senior data clerk. The senior data expert worked with the senior data clerk to conduct the data entry, cleaning, and analysis.

#### Data analysis

The data were analyzed by age, sex, TB classification, TB category, treatment outcome, and place of residence. Patients were classified into two groups based on their place of residence: those from Adama City and those from outside the city. The CNR was calculated by the number of TB cases notified per 100,000 population. The treatment success rate was calculated by dividing the number of TB cases cured or who completed treatment by the total number of TB cases registered for treatment. Case finding and treatment outcomes were calculated based on the standard definition in the national guidelines. No report was generated based on place of residence, however.

#### Ethical considerations

Because this study was a retrospective review of data gathered under routine program implementation as a public health practice, ethical approval was not required as no personally identifiable data were collected, so the privacy and confidentiality of patients were assured.

## Results

### Tuberculosis case finding

A total of 2892 TB cases were registered at health facilities providing TB services in Adama City. Of these, 2432 (84%) TB cases were from Adama City, while 460 (16%) were from other catchment areas (Table 1). Of 2432 TB cases, 46% of the TB cases were females and 60% were in the age range of 25–64 years. The mean age was 31 years (SD + 14.5). Of the total number of cases, 34% (981) were bacteriologically confirmed (PPOS), 29% (822) were pulmonary negative (PNEG), and 37% (1072) were extrapulmonary (EPTB) cases. Of the TB cases, 95% of the TB cases were newly diagnosed, and 7% were children under the age of 15 years.

**Table 1:** TB cases notified in Adama City, 2014/15–2017/18

TB Case Finding^a^	PPOS^b^	PNEG^c^	EPTB^d^	Total
Within Adama	801	691	928	2420
	82%	84%	87%	84%
Outside Adama	180	131	144	455
	18%	16%	13%	16%
Total	981	822	1072	2875^1^
	100%	100%	100%	100%

^a^17 TB cases were not included because the patients’ addresses were missing ^b^PPOS: pulmonary smear positive TB cases ^c^PNEG: clinically diagnosed smear negative pulmonary TB cases ^d^EPTB: extra pulmonary TB cases

The total or unadjusted TB CNR (including TB cases registered from the Adama catchment population and nearby catchment population) varied between 153 and 218 per 100,000 population over the 1 years of the study. However, the adjusted TB CNR (excluding TB cases outside of Adama City) fell between 135 and 179 per 100,000 population. On average, about 16% of the TB cases notified by the Adama City TB program were not from the Adama catchment population (Table 2).

**Table 2:** Trends in TB case notification in Adama City, 2014/15–2017/18

Year	2014/15	2015/16	2016/17	2017/18
Total population	323,999	338,940	355,475	372,817
TB cases treated in Adama City	707	760	762	569
TB cases living in Adama City	581	604	667	502
% TB cases living in Adama city	82.2%	79.5%	87.5%	88.2%
CNR (*per 100,000 population*)*				
TB cases enrolled in Adama City	218	224	214	153
TB cases living in Adama City	179	178	188	135
TB cases living outside Adama City	39	46	26	18

* X^2^ = 12, *P*-value = 0.213

**Fig. 1:**
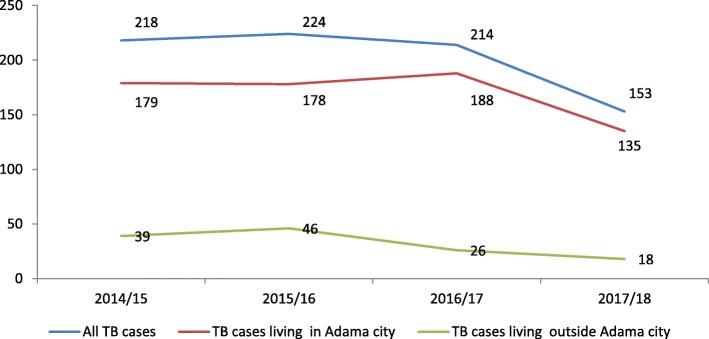
Trends of TB case notification rate in Adama City, 2014/15–2017/18

The CNR of 32 per 100,000 population (ranging from 18 to 46 per 100,000) for Adama City was from the patients that came from the surrounding rural areas who sought care in the town (Fig. 1).

### Tuberculosis treatment outcomes

Of 1782 TB cases registered, data about treatment outcome were available for 1737 (97%) cases. The treatment success rate was 95% both from outside and within Adama City; i.e., 65% completed treatment (65% from Adama City and 61% from outside) and 30% were cured (28% from Adama City and 34% from outside). There is no statistically significant difference in treatment outcome among patients, whether they reside within Adama City or not (*P* = 0.45) (Tables 3 and 4).

**Table 3:** Treatment outcomes of TB cases in Adama City, 2014/15–2017/18

Treatment outcome	PPOS^a^	PNEG^b^	EPTB^c^	Total
Cured^*^	515			515
	88%			30%
Treatment completed^*^	52	507	612	1137
	9%	95%	94%	65%
Treatment failure	2	3	8	13
	0%	1%	1%	1%
Died	9	20	23	52
	2%	4%	4%	3%
Lost to follow-up	3	6	5	14
	1%	1%	1%	1%
Moved to MDR-TB register	4	0	2	6
	1%	0%	0%	0%
Total	585	536	650	1737
	100%	100%	100%	100%

^a^*PPOS* Pulmonary smear positive TB cases ^b^*PNEG* Clinically diagnosed sear negative pulmonary TB cases ^c^*EPTB* Extra pulmonary TB cases

^*^The mean treatment success rate (cure rate d + treatment competed rate) of PPOS cases was 97% (SE = 0.007) and PNEG was 95% (SE = 0.009), Z = 1.72 *p*-vale = 0.086 with no statistical significance. In comparison with EPTB compare to EPTB with treatment success rate of 94% 9SE = 0.009), Z = 2.52, *p*-value = 0.012 which is statistically significant

**Table 4:** Treatment outcomes of TB cases treated in Adama City, 2014/15–2017/18

Treatment Outcome^a^		Outside Adama	Inside Adama	Total
Cured	No.	98	417	515
	%	34%	28%	30%
Treatment completed	No.	175	962	1137
	%	61%	65%	65%
Treatment failure	No.	2	11	13
	%	1%	1%	1%
Died	No.	8	44	52
	%	3%	3%	3%
Lost to follow-up	No.	4	10	14
	%	1%	1%	1%
Moved to MDR-TB^b^ register	No.	2	4	6
	%	1%	0%	0%
Total	No.	289	1484	1737
	%	100%	98%	100%

Treatment success rate was 95% (34% cured and 61% treatemen comlplted) for those from outside Adama and 93% (28% cured and 65% treatemen comlplted) for those from inside Adama city, with no statistical significance, *p* = 0.45

^a^Comparison of treatment outcome was not statistically signficant, X^2^ = 35, *p*-value = 0.243

^b^*MDR-TB* Multi drug resistant tuberculosis

## Discussion

In this study, we report high TB CNRs in Adama City. However, about one-fifth of the TB cases notified were contributed by patients from outside the catchment population. This overstates the urban CNR and underestimates the CNR of the neighboring catchment areas. However, disaggregating the data about TB cases by place of residence offset the high CNR in urban and low CNR in adjacent communities, which could give the real picture of TB case notification in the areas. Like other studies in urban populations [19–21], studies from Ethiopia show that the urban population, representing only 8% of the country’s population, contributed 11% of the total TB cases notified [22]. This phenomenon could be due to patients coming from neighboring catchment areas for access to better diagnostics and availability of technical expertise, thereby increasing urban poverty, overcrowding, urban migration, HIV infection, and disease transmission [9]. This pattern of care seeking might have contributed to the increasing focus on urban TB programs.

The Zero TB Cities Initiative is one of the major efforts to combat TB and its transmission [6]. Such efforts, however, should consider detailed analysis of case finding in urban areas by place of residence, which is not commonly done [22]. Without specific analysis with regard to place of residence, the CNR of adjacent rural districts may be underestimated and the urban CNR may be inflated. Thus, high CNRs may give the impression that targets are being met in urban areas and may lead to reluctance to target TB in surrounding areas by urban TB programs. Inadequate data can, in turn, affect resource allocation. An increase in resources for urban TB programs could undermine efforts to reach surrounding catchment areas, which could contribute to continued disease transmission in peri-urban areas.

Evidence has shown that disaggregation of data about notified TB cases by urban or rural place of residence has reduced the CNRs of areas that were known for higher CNRs and increased the CNRs of areas that did not report many cases. A ten-year review of TB cases notified by districts in southern Ethiopia showed that about 23% of the TB cases notified came from other catchment areas or districts. The disaggregation of TB cases by their residence reduces the high CNRs of urban areas and increases the lower CNRs in adjacent rural areas [23].

Higher CNRs could be driven by patient preference, better service quality, increased geographic accessibility, better community awareness, and access to better diagnostics and treatment [23]. Analysis of CNR by place of residence offsets both under- or over-reporting in urban and rural communities. Failure to consider this reality may contribute to urban bias, with the possible resource implications mentioned above, and may affect the type and magnitude of interventions designed by NTPs. Therefore, interventions in urban settings should analyze cases by place of residence, consider factors underlying higher CNRs, and design appropriate interventions to reach TB cases missed in the urban population.

While there are clear justifications for prioritizing TB in urban areas, other factors should be considered to ensure efficient use of scarce resources. In most resource-limited settings, a significant portion of urban health-service seekers come from rural areas, sometimes travelling long distances, due to lack of quality health services in remote areas. In addition, frequent bidirectional movements of people between urban and rural areas [24] for various purposes may increase disease transmission. Since most project-driven TB case-finding efforts use accessibility, feasibility, and yield as criteria for selection of intervention sites, there is a high probability that remote, rural, and low-case-notifying areas will be left behind. This issue suggests the need for review of the deceptively high CNRs in urban areas, so that efforts to reach remote areas or areas with low CNRs are not undermined. Urban areas, with their higher populations and compromised socioeconomic conditions, require interventions to strengthen the networking of urban-rural programs to reach their actual catchment populations in order to improve case finding. Ending TB will only be possible if the urban rural disaggregation of data leads designing interventions and reaching missed cases whether they are not diagnosed which will remain to be source of continued transmission or diagnosed and not notified by the health system.

In the absence of subnational TB prevalence reports, TB investment and the performance of TB programs should be evaluated in the light of actual CNRs, using data about patients’ place of residence as well as their place of treatment. This analysis will ensure the use of accurate data for decision-making and action. Further analysis of program limitations related to referral linkages, treatment success, and program capacity to reach target populations is needed.

Failure to account for patients from Adama City who might have sought care in surrounding rural areas could have led to underestimating the CNR in urban areas, and inability to verify the address registered, whether it is place of residence or temporary address, might have affect the results. Our study is limited to Adama city and did not measure the case notification and treatment success of the surrounding population which could give better estimate of the contribution of rural communities and identify TB cases from urban who received treatment in the rural. Further study is required to understand the patient flow between urban and rural areas to estimate CNRs in such settings.

This study could be generalized to urban sites were TB patients receive services within the catchment population and receive patents from surrounding areas that could increase the reported case notification of urban sites. In areas where patients are not strictly receiving treatment within their place of residence such underestimation of the real picture of the surrounding sites could be noticed while the urban areas could have higher notification rate which could be misleading. The results of the study should be cautiously interpreted as the sample size across the two groups was small to pick statistical difference among the two groups.

## Conclusion

Urban TB program managers need to understand that the number of TB cases notified from urban areas includes cases from surrounding sites. Therefore, NTPs should analyze urban TB data by disaggregating the data by place of residence to identify gaps in case finding and strengthen urban case finding to reach missed or unreached cases in urban communities. Larger studies are warranted to address the needs of key populations in urban areas.

## Data Availability

The datasets used and/or analyzed in this study is available from the corresponding author on reasonable request.

## Abbreviations

CNR: Case Notification rate
EPTB: Extra pulmonary Tuberculosis
HIV: Human Immunodeficiency Virus
NTP: National Tuberculosis Program
PNEG: Pulmonary Smear Negative Tuberculosis
PPOS: Pulmonary Smear Positive Tuberculosis
TB: Tuberculosis
